# Akzeptanzanalyse von Microsoft Teams als eKollaborationssystem bei standortverteilten und agilen Entwicklungsteams eines mittelständischen Unternehmens

**DOI:** 10.1365/s40702-021-00776-2

**Published:** 2021-08-23

**Authors:** Jonathan Schwind, Fahri Yetim

**Affiliations:** FOM Hochschule für Oekonomie & Management, Hochschulzentrum, Agrippinawerft 4, 50678 Köln, Deutschland

**Keywords:** Technologieakzeptanz, Microsoft Teams, Kollaborationssysteme, Verteilte Systementwicklungsteams, Technology acceptance, Microsoft Teams, Collaboration systems, Distributed system development teams

## Abstract

eKollaborationssysteme haben das Ziel, die Zusammenarbeit über zeitliche und räumliche Grenzen hinweg zu unterstützen. *Microsoft Teams* hat sich mittlerweile als ein eKollaborationssystem etabliert. Bisherige Forschung unterstreicht im Allgemeinen die Relevanz der Benutzerakzeptanz für die erfolgreiche Einführung und Nutzung von eKollaborationssystemen in Unternehmen. Jedoch mangelt es an empirischen Akzeptanzstudien, die sich spezifisch mit der Akzeptanz von *Microsoft Teams* beschäftigen. Diese Arbeit untersucht die Akzeptanz des Einsatzes von *Microsoft Teams* zur Kommunikation in standortverteilten agilen Entwicklungsteams im Kontext eines mittelständischen Unternehmens. Anhand einer Fallstudie sollen Erkenntnisse über Akzeptanz- und Nutzungsverhalten eines Entwicklungsteams im konkreten praktischen Anwendungskontext gewonnen werden, um daraus praktische Handlungsempfehlungen zur zielgerichteten Optimierung des Systems und dessen Einsatz für die Entscheidungsträger abzuleiten. Zu diesem Zweck wird eine qualitative empirische Vorgehensweise gewählt, die sich an Akzeptanztheorien orientiert, um die wichtigsten Einflussfaktoren der Akzeptanz von Microsoft Teams theoriegeleitet verstehen und erklären zu können. Die Ergebnisse bestätigen unter anderem, dass die Leistungserwartung und Aufwandserwartung besonders relevant in diesem Zusammenhang sind. Die Implikationen der Ergebnisse für weitere Forschung und Praxis werden ebenfalls kurz vorgestellt.

## Abstract (en)

The purpose of e‑collaboration systems is to support collaboration across time and space boundaries. *Microsoft Teams* has now established itself as an e‑collaboration system. Research to date generally underlines the relevance of user acceptance for the successful introduction and use of e‑collaboration systems in companies. However, there is a lack of empirical acceptance studies that specifically deal with the acceptance of *Microsoft Teams*. This work examines the acceptance of the use of *Microsoft Teams* for communication in agile development teams distributed across locations in the context of a medium-sized company. Based on a case study, insights into the acceptance and usage behavior of a team in a specific practical application context are to be obtained in order to derive practical recommendations for the targeted optimization of the system and its use for the decision-makers. For this purpose, a qualitative empirical method is chosen that is based on acceptance theories, in order to be able to understand and explain the most important factors influencing the acceptance of *Microsoft Teams* in a theory-guided manner. The results confirm, among other things, that the performance expectation and the effort expected are particularly relevant in this context. The implications of the results for further research and practice are also briefly presented.

## Einleitung

Die Zusammenarbeit über zeitliche und räumliche Grenzen hinweg prägen zunehmend die Arbeitsrealität von Teams in und zwischen Unternehmen in einer globalisierten und vernetzten Welt (Boos et al. 2017; Lindner 2020). Vor allem in wissensintensiven Bereichen wie der Softwareentwicklung findet die Zusammenarbeit in geographisch verteilten Entwicklungsteams statt. Im besonderen Maße erfordern agile Arbeitsmethoden flexible Reaktionen auf Veränderungen, Demokratisierung der Arbeitsorganisation und die Stärkung der Kommunikation etc., um eine effektive und effiziente Zusammenarbeit zu ermöglichen. eKollaborationssysteme haben das Ziel, die Zusammenarbeit zu unterstützen (Riemer 2009; Schrauzer 2018; Riemer et al. 2019). Eine große Herausforderung für Unternehmen ist derzeit, die Kontrolle über den Datenfluss innerhalb der verschiedenen Applikationen zu behalten und einheitlich integrierte Gesamtlösungen zu implementieren, um geregelte Kommunikation sicherzustellen. Auf Basis der Befragung von 1465 Mitarbeitern aus Deutschland, Österreich und Schweiz kommt die „Deutsche Social Collaboration Studie 2019“ unter anderem auch zu dem Ergebnis, dass zwei Drittel der Befragten mit der Einführung der Social-Collaboration-Tools nicht zufrieden sind (Buxmann 2019, S. 3).

Auch das schweizerische Softwareunternehmen Cuvox AG (Firmenname aus Datenschutzgründen pseudonymisiert) steht mit mehr als 400 Mitarbeitern an über acht Standorten in der Schweiz, Deutschland und Kroatien vor der umfassenden Aufgabe, die Produktivität der Entwicklungsteams bei zunehmend erhöhter standortverteilter Zusammenarbeit zu steigern. Beinahe die gesamte agile Softwareentwicklung arbeitet mit standortverteilten *Scrum-Teams*. Die Unternehmensorganisation wurde in den vergangenen Jahren in vielen Bereichen auf eine Zusammenarbeit zwischen den Standorten ausgerichtet. Dazu zählt die Bereitstellung der Infrastruktur, Lizenzrechte und Integration von Anwendungssystemen für Telefonie und Instant-Messaging Diensten wie *Skype for Business* oder *Slack*. Strategische Entscheidungen zielen mit der Einführung von *Microsoft 365* und des Kollaborationssystems *Microsoft Teams* auf eine einheitliche Anwendungssoftware ab.

Bisherige Forschung unterstreicht im Allgemeinen die Relevanz der Benutzerakzeptanz für die erfolgreiche Einführung und Nutzung von eKollaborationssystemen in Unternehmen (Venkatesh und Davis 2000; Benke und Maedche 2019; Buxmann 2019). Jedoch mangelt es an empirischen Akzeptanzstudien, die sich spezifisch mit der Akzeptanz von *Microsoft Teams* beschäftigen. Diese Arbeit untersucht die Akzeptanz des Einsatzes von *Microsoft Teams* zur Kommunikation in standortverteilten agilen Entwicklungsteams im Kontext des mittelständischen Softwareunternehmens Cuvox AG. Anhand einer Fallstudie sollen Erkenntnisse über Akzeptanz- und Nutzungsverhalten eines Teams im konkreten praktischen Anwendungskontext gewonnen werden, um daraus praktische Handlungsempfehlungen zur zielgerichteten Optimierung des Systems und dessen Einsatzes für die Entscheidungsträger abzuleiten. Zu diesem Zweck wird eine qualitative empirische Vorgehensweise gewählt, die sich an Akzeptanztheorien orientiert, um die wichtigsten Einflussfaktoren der Akzeptanz von *Microsoft Teams* theoriegeleitet verstehen und erklären zu können. Im Folgenden stellen wir zunächst die theoretischen Grundlagen der Arbeit und den Stand der Forschung dar und präsentieren anschließend die Methodik sowie auch die Ergebnisse. Abschließend erfolgt eine Diskussion der Ergebnisse und Implikationen für Forschung und Praxis.

## Theoretische Grundlagen und Stand der Forschung

### Modelle zur Erklärung der Technologieakzeptanz

In der Literatur existiert eine Vielzahl von Modellen, die die Akzeptanz und Nutzung von Technologien erklären. Das erste *Technology Acceptance Model* (TAM) (Davis et al. 1989) betrachtete zwei Variablen als fundamentale Determinanten der Akzeptanz: die *wahrgenommene Nützlichkeit* und die *wahrgenommene Einfachheit der Nutzung*. Beide Determinanten wirken auf die Nutzungseinstellung (Nutzungsabsicht), welche die tatsächliche Nutzung des Systems beeinflusst. Im Laufe der Jahre wurde dieses Modell mehrfach erweitert. Im TAM 2 (Venkatesh und Davis 2000) wurden zusätzlich Faktoren berücksichtigt, die auf die *wahrgenommene Nützlichkeit* von Systemen einwirken, wie z. B. die sozialen Einflussfaktoren (Soziale Normen und Image) und kognitive Prozesse (Arbeitsplatzrelevanz, Ergebnisqualität und vorzeigbare Ergebnisse). Im TAM 3 (Venkatesh und Bala 2008) sind auch Faktoren integriert, die auf die *wahrgenommene Einfachheit der Nutzung *von Systemen einwirken, u. a. die Wahrnehmung der externen Kontrolle, die Angst vor Computer, die Computerbezogene Spielfreude, das wahrgenommene Vergnügen.

**Abb. 1:**
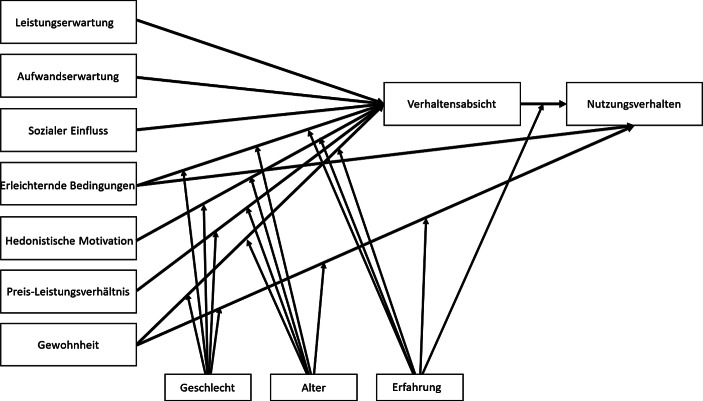
Einheitliche Theorie der Akzeptanz und Nutzung von Technologie (UTAUT 2). (Quelle: In Anlehnung an *Venkatesh et* *al.* (2012), S. 160)

Parallel zu dieser Entwicklung entstand auch eine vereinheitlichte Technologie-Akzeptanz- und Nutzungstheorie, i.e. *Unified Theory of Acceptance and Use of Technology *(UTAUT), die verschiedenen Theorien und Vorgängermodelle der Akzeptanzforschung integrierte (Venkatesh et al. 2003). Die erste Version des Modells bezog sich primär auf einen organisationalen Kontext und berücksichtige vier Konstrukte als wesentliche Determinanten, welche die *Verhaltensabsicht* und das *Nutzungsverhalten* beeinflussen: *Leistungserwartung* (analog wahrgenommene Nützlichkeit), *Aufwandserwartung* (analog wahrgenommene Einfachheit der Nutzung), *Sozialer Einfluss* und *Unterstützende Bedingungen* in Organisationen. In der Erweiterung des Modells UTAUT‑2 (Venkatesh et al. 2012) wurden zum einen zusätzlich drei Konstrukte *hedonische Motivation, Preis-Leistungsverhältnis* und *Gewohnheit* eingeführt, um den Consumer-Kontext zu berücksichtigen, und zum anderen Wirkungsbeziehungen abgeändert (vgl. Abb. 1).

Diese Arbeit orientiert sich an den Konstrukten des UTAUT-2-Modells, die daher hier kurz beschrieben werden. Die *Leistungserwartung* gilt als Maß für die Überzeugung des Nutzers, dass die Nutzung der Technologie positiv auf die Arbeitsleistung auswirkt. Dieser wahrgenommene Nutzen hat den stärksten Einfluss auf die Verhaltensabsicht. Die *Aufwandserwartung* gibt an, inwieweit der Nutzer die Nutzung der Technologie als aufwendig einstuft. Meistens ist damit zeitlicher oder kognitiver Aufwand gemeint. Der *soziale Einfluss* bezieht sich darauf, inwieweit der Nutzer von seinem sozialen Umfeld (Normen, Werte und Bezugspersonen) beeinflusst wird. Die *hedonistische Motivation *gibt an, inwieweit der Nutzer Freude bzw. Spaß bei der Nutzung der Technologie empfindet. *Das Preis-Leistungsverhältnis* zeigt die Beurteilung der Differenz zwischen dem Kosten (Preis) und Nutzen (wahrgenommenem Mehrwert) der Technologie an. Im Kontext der Verbraucherakzeptanz hat dieses Konstrukt einen großen Einfluss auf die Verhaltensabsicht (bzw. Nutzungsintention), vor allem wenn der Verbraucher die Nutzungskosten selbst tragen muss. Schließlich gibt das Konstrukt der *Gewohnheit* den Grad an, zu dem Nutzer ein bestimmtes Nutzungsverhalten, welches sie in der Vergangenheit erlernt haben, automatisch ausführen.

### Microsoft-Teams als eKollaborationssystem

#### eKollaboration und eKollaborationssysteme

Technische Unterstützung der Kommunikation und Zusammenarbeit in Teams und Projekten in und zwischen Unternehmen wird in einer zunehmend globalisierten und vernetzten Welt zur Grundvoraussetzung effektiven Arbeitens (Riemer 2009). Kleine und interdisziplinäre aufgestellte Entwicklungsteams, die innerhalb kurzer und eng mit dem Kunden abgestimmten Entwicklungszyklen, Produktinkremente ausliefern können, sind ein zentraler Erfolgsfaktor von agilen Organisationen. Die internetbasierte Zusammenarbeit der Mitglieder der Teams an unterschiedlichen Orten und in unterschiedlichen Zeiten, wird als eKollaboration bezeichnet. eKollaboration bezieht sich auf die Vorgänge und Prozesse der Kommunikation, Koordination und Kooperation zwischen Menschen in verteilten Projekten, Prozessen und Teams in und zwischen Organisationen mittels Informations- und Kommunikationstechnologie (Riemer 2009; Döbler 2019; Benke und Maedche 2019).

Systeme, die diese Prozesse unterstützen, werden als eKollaborationssysteme oder -Technologien bezeichnet. eKollaborationssysteme stellen vielfältige Funktionen zur Unterstützung bzw. Ermöglichung der kollaborativen Zusammenarbeit. Zu den Kommunikationsfunktionen gehören z. B. Emails, Chats, Audio- und Videokonferenzen, Diskussionsforen, Weblogs. Typische Funktionen der Koordinationsunterstützung beziehen sich auf die Termin‑, Prozess- und Aufgabenkoordination in Projekten, und unterstützen die Abstimmung der Teammitglieder untereinander. Kollaborative Zusammenarbeit schließlich ist durch Kooperation und gemeinsame Ziele gekennzeichnet. Einige Funktionen von eKollaborationssystemen (wie z. B. Gruppeneditoren, Wikis, elektronisches Whiteboard, Application Sharing) unterstützen die Zusammenarbeit an der gemeinsamen Erstellung und Bearbeitung von Objekten bzw. Dokumenten. Dabei finden sich im Markt sowohl einfache Tools als auch zunehmend integrierte Systeme, die je nach Ausrichtung eine Vielzahl von Funktionen bieten, die für die Zusammenarbeit relevant sind (Riemer 2009; Lindner 2020).

#### Microsoft Teams als eKollaborationssystem

*Microsoft* bietet verschiedene Produkte zur eKollaboration. Die Produkte *Microsoft Office 365 *und *Microsoft Teams* kombinieren diverse Online-Services mit der klassischem Desktop-Office Software, einschließlich Anwendungen wie Schreibprogramm (*Word), *Tabellen *(Excel)*, Präsentationen (*PowerPoint)*, Notizen (*OneNote*), Videokonferenz und Chatsystem (*Skype*), E‑Mail (*Outlook*) und Filesharing (*OneDrive)* (Microsoft 2021). *Microsoft Teams* wurde im Jahr 2017 veröffentlicht und enthält zusätzliche Funktionen für den Bereich Nachrichten, Meeting und Aufgabenverwaltung (Lindner 2020) und bedient mit seinen Funktionen die drei Felder Kommunikation, Koordination und Kollaboration.

Die Kommunikation für mehrere, einer Gruppe (Team) zugehörigen Personen erfolgt über die jeweiligen Kanäle. Über diese Kanäle können die Team-Mitglieder Chat-Nachrichten oder Dateien austauschen oder Besprechungen *ad hoc* durchführen. Kontakte können auch aus der Chat-Funktion mittels Audio- oder Videoanruf kontaktiert werden. Es wird unterschieden zwischen Einzelchats, Gruppenchats und Chats für Meetings. Dokumente oder Dateien können auch von mehreren Personen gleichzeitig bearbeitet werden, sofern eine Integration des Speicherdienstes von Microsoft (*OneDrive for Business*) besteht.

### Studien zur Akzeptanz von eKollaborationssystemen und Microsoft Teams

Die Literaturrecherche zeigt, dass es vereinzelte Studien zur Akzeptanz von kollaborativen Systemen gibt. Die meisten Studien beziehen sich jedoch nicht auf den Einsatz von Systemen zur Unterstützung der Zusammenarbeit, sondern auf das Thema kollaboratives Lernen und verwenden ältere Akzeptanzmodelle (vgl. Pal und Vanijja 2020). Darüber hinaus existieren Studien, die sich allgemein mit empirischen Befragungen zur Produktivität und Zufriedenheit der Mitarbeiter bei der Nutzung neuer Kommunikationstechnologien beschäftigen, ohne auf die verwendeten eKollaborationssysteme der Befragten einzugehen (Vgl. Greisle 2004). Auch die „Deutsche Social Collaboration Studien“ geben jährlich durch Befragungen in Unternehmen aus Deutschland, Österreich und der Schweiz einen toolunabhängigen Gesamtüberblick über die Nutzung moderner Technologien zur Förderung der vernetzten Zusammenarbeit. Die Studie von 2019 (Buxmann 2019) berücksichtigt Szenarien, die den wichtigsten Aktivitäten der alltäglichen Kommunikation und Zusammenarbeit in Unternehmen entsprechen, wie z. B. dem Austausch von Dokumenten oder der Suche nach einem Experten. Die Teilnehmer der Studie wurden bei jedem Szenario gefragt, inwieweit sie dafür aktuelle digitale Technologien verwenden. Die Kernaussagen der Studie 2019 (Buxmann 2019, S. 3) zeigen, unter anderem, dass zwei Drittel der Befragten mit der Einführung von *Social Collaboration Tools* nicht zufrieden sind, und dass durch Change-Management-Maßnahmen mehr Berücksichtigung der Bedürfnisse der Mitarbeiter und ausreichend Zeit für die Mitarbeiter zur Eingewöhnung an die Tools erwünscht sind.

Da diese Studien die verwendeten konkreten Kollaborationssysteme nicht berücksichtigen, können hieraus keine Aussagen über bevorzugte Systeme getroffen werden. Ein Vergleich zwischen den Kollaborationssystemanbietern *Slack* und *Microsoft Teams* zeigt, dass *Microsoft Teams* bis November des Jahres 2019 die aktiven Nutzerzahlen weltweit auf 20 Mio. erhöht hat und deutlich vor *Slack* (12 Mio.) liegt (Statista 2019). Diese Kennzahlen haben jedoch geringe Aussagekraft, da sie zum einen nicht darüber informieren, ob diese Systeme nur zu eKollaboration genutzt oder überwiegend als Videokonferenzsystem eingesetzt wurden. Zum anderen legen diese Kennzahlen keine Erkenntnisse über die Faktoren, die die Wahrnehmung und Akzeptanz der Nutzer beeinflussen, und über ihre subjektiven Auswirkungen offen. Folglich lassen sich hieraus keine Ansatzpunkte für eine Steuerung bzw. Optimierung der Akzeptanzfaktoren ableiten. Unsere Literaturrecherche mit *Google Scholar* lieferte keine Studie zur Akzeptanz des Einsatzes von* Microsoft Teams* als eine eKollaboration-Plattform. Ausgehend von der Problematik der mangelnden Erkenntnisse über die Nutzung und Akzeptanz von *Microsoft Teams *besteht das Ziel der vorliegenden Arbeit darin, den Einsatz von *Microsoft Teams *in Kontext eines verteilt arbeitenden Teams in der agilen Anwendungsentwicklung zu untersuchen. Im Vordergrund stehen dabei vor allem die Beantwortung folgender Fragen: Wie gehen Mitarbeiter mit den Funktionen von *Microsoft Teams* um? Welche Funktionen werden akzeptiert bzw. nützlich erachtet, welche nicht? Welche zukünftige Akzeptanzeinstellung hat sich gegenüber *Microsoft Teams* etabliert? Welche Handlungsempfehlungen lassen sich daraus ableiten?

## Methode

Die Beantwortung der relevanten Forschungsfragen erfordert ein vertieftes und umfassendes Verständnis des Untersuchungsgegenstandes aus Perspektiven des Untersuchungssubjektes. Im Folgenden wird die zu diesem Zweck gewählte qualitative empirische Methode (Mayring 2015) mit Fokus auf die wesentlichen Aspekte der Datenerhebung und der Auswertung dargestellt. Nähere Details zur Vorgehensweise und Methode finden sich in Schwind (2020).

### Datenerhebung

Für die Datenerhebung wurden acht Mitglieder eines verteilten Entwicklungsteams der Cuvox AG interviewed. Die Mitglieder des Teams arbeiteten während der Datenerhebung sowohl in Deutschland als auch in der Schweiz aufgrund der Covid-19-Pandemie mehrheitlich im Home-Office. Das Team hatte bereits Erfahrungen mit anderen Kollaborationssystemen und nutzte *Microsoft Teams* während der sechsmonatigen Testphase in ihrem Arbeitsumfeld für die teaminterne Kommunikation, einschließlich mehrerer Team-Termine wie Daily Scrum, Review oder Retrospektive, welche über Videokonferenzen abgehalten wurden. Das Entwicklungsteam verfolgte das Konzept der agilen Softwareentwicklung nach *Scrum*, mit einer Rollenverteilung im Entwicklungsteam: Softwareentwickler (8), Software Tester (1), Agile Writer (1), Product Owner (1), Scrum Master (1).

Für das Interview wurde ein teilstrukturierter Interviewleitfaden nach Helfferich (2019) entwickelt. Dafür wurde zuerst die Literatur zur Technologieakzeptanz analysiert, um grobe Anhaltspunkte für die deduktiven Kategorien zu sammeln. Anschließend wurde mit der Brainstorming-Methode eine Vielzahl von Fragen gesammelt. Diese Fragen wurden darauf geprüft, ob sie zielführend für die Beantwortung der Forschungsfrage sind, und ob sie offene, explorative Antworten zulassen. Nach der Elimination der irrelevanten Fragen wurden die Fragen in Haupt- und Nebenfragen sortiert und zu unterschiedlichen Fragenblöcken subsummiert, unter Beachtung ihrer logischen und zeitlichen Abfolge. Die Haupt- und Nebenfragen, die in den Kontext eines verteilt arbeitenden Teams in der agilen Anwendungssystementwicklung gesetzt wurden, thematisieren vor allem die Vorerfahrungen der interviewten Personen mit eKollaborationssystemen, ihren aktuellen Umgang mit *Microsoft Teams *und ihre Wahrnehmung bzw. Bewertung der einzelnen Funktionen des Systems sowie auch ihre zukünftige Nutzungsabsicht. Die Fragen wurden offen gestellt, um ein breites (exploratives) Antworten zu ermöglichen und induktiv weitere Faktoren zu ermitteln. Der Interviewleitfaden wurde in einem Pretest erprobt und nochmals angepasst.

Die leitfadengestützten Interviews wurden im Zeitraum von August bis Oktober 2020 durchgeführt. Die Teammitglieder wurden nach ihrer zeitlichen Verfügbarkeit ausgewählt und per E‑Mail benachrichtigt. Sie wurden mit weiteren Informationen zum Hintergrund der Befragung sowie auch zu Datenschutzmaßnahmen versorgt. Aufgrund der Covid-19-Pandemie wurden die Interviews teilweise über *Skype for Business* als auch über *Microsoft Teams* durchgeführt. Jedes Interview dauerte im Durchschnitt ca. 35 min. Die Interviews wurden in Bild und Ton aufgezeichnet und anschließend transkribiert. Die Interviews, Transkription von Interviews und die erste Auswertung der Daten wurden vom ersten Autor durchgeführt, der über Kennnisse im Fachgebiet agile Anwendungsentwicklung verfügt und mit dem Arbeitskontext der Cuvox AG vertraut ist. Zur Transkription und Auswertung der Daten wurde die Software MAXQDA verwendet.

### Datenauswertung

Die Auswertung von Interviews orientierte sich an der strukturierenden qualitativen Inhaltsanalyse nach Mayring (2015), dessen Ziel es ist, das Forschungsmaterial anhand vorher definierter Kriterien zu untersuchen und einzuschätzen. Hierzu handelt es sich um eine *deduktive Kategorienanwendung*, bei der ein Kategoriensystem vorab theoriegeleitet entwickelt und dann an den Text herangetragen wird.

Das in dieser Arbeit verwendete Kategoriensystem wurde durch die Zusammenstellung der Hauptkategorien aus dem Akzeptanzmodell UTAUT‑2 gebildet und zur kategorienbasierten Auswertung angewandt. Um eine eindeutige Zuordnung der Analyseeinheiten (Sätze, Antworten auf Interviewfragen) zu unterstützen, wurden die Definitionen der Kategorien und Ankerbeispiele in einem Kodierleitfaden definiert (Mayring 2015). Zusätzlich wurden sowohl deduktive Unterkategorien aus UTAUT-Modell als auch induktive Kategorien, die aus dem Material selbst erstellt wurden, berücksichtigt.

Das Kategoriensystem enthält 12 Hauptkategorien: *Leistungserwartung, Aufwandserwartung, sozialer Einfluss, erleichternde Bedingungen, hedonistische Motivation, Gewohnheit, Verhaltensabsicht* und *Nutzungsverhalten*. Die Kategorie *Preis-Leistungsverhältnis* des UTAUT-2-Modells wurde in dieser Studie nicht verwendet, da das Entwicklungsteam keine Informationen über die Kosten von *Microsoft Teams* bei der Cuvox AG hat und somit keine Auswirkung dieser Variable erwartet wird.

**Abb. 2:**
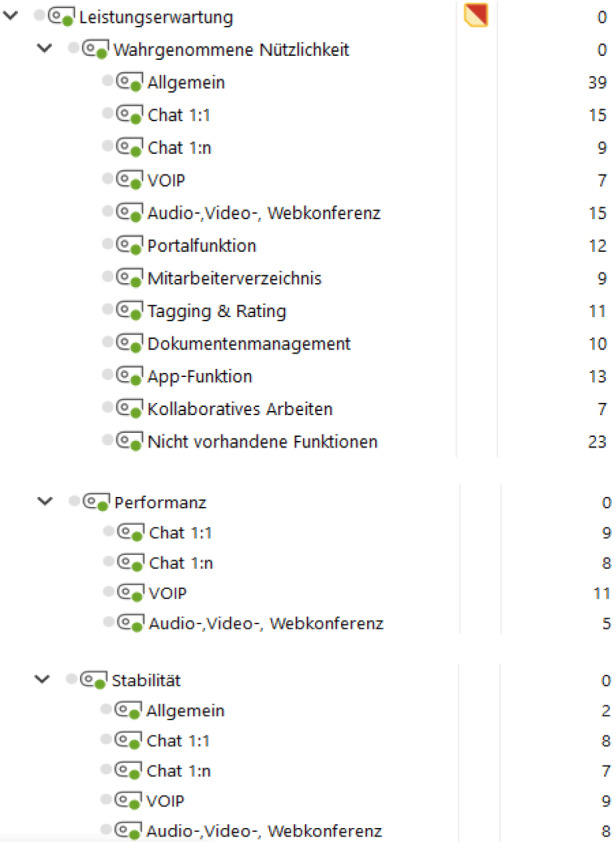
Kodierte Einheiten zu Unterkategorien der Leistungserwartung

Die Abbildung (Abb. 2) stellt exemplarisch einen Ausschnitt der Kodierungsergebnisse dar und zeigt die kodierten Einheiten zu den Unterkategorien der Hauptkategorie *Leistungserwartung*, die sich auf die einzelnen Funktionen von Microsoft Teams beziehen. Nähere Details zum Kodierungsleitfaden und zu gesamten Kodierungsergebnissen finden sich in Schwind (2020).

Abschließend soll über die Qualität der Vorgehensweisen in dieser qualitativen Studie kurz reflektiert werden. Erstens: Die Strategie zur Geltungsbegründung bezieht sich in dieser Arbeit auf die Transparenz der Vorgehensweisen (Flick 2019). In diesem Beitrag wurden zu diesem Zweck die wesentlichen methodischen Schritte für die Datenerhebung und Auswertung kurz dargestellt und für weitere Details zu methodischen Entscheidungen und zwischen Ergebnissen auf die ausführliche und nachvollziehbare Dokumentation in Schwind (2020) verwiesen. Zweitens: Die durch den ersten Autor durchgeführte Auswertung der Daten wurde vom zweiten Autor mit fachlichem Hintergrund im Bereich Technologieakzeptanzforschung überprüft, woraus im Dialog die abschließende Interpretation der Ergebnisse zum Zwecke der Publikation entstand. Dies entspricht etwa der Vorgehensweise zur „*Validierung von Ergebnissen durch andere Forschende“*, denen sie zur Kommentierung und Bewertung vorgelegt werden (Flick 2019, S. 477). Drittens: Zum Zwecke der intersubjektiven Überprüfbarkeit wird darauf Wert gelegt, durch die folgende verständliche Darstellung der Ergebnisse und der anschließenden Diskussionen, die Reflektionen, Schlussfolgerungen bzw. Verallgemeinerungen aus den Ergebnissen offen zu legen.

## Ergebnis

Im Folgendem werden die Ergebnisse im Zusammenhang mit den Kategorien des UTAUT-2-Modells zusammenfassend kurz vorgestellt.

### Leistungserwartung

Die Kategorie der Leistungserwartung bezieht sich auf die von den Benutzern erwarteten Leistungen des Systems und besteht aus drei Unterkategorien: wahrgenommene Nützlichkeit, Performanz und Stabilität (vgl. Abb. 2). In Bezug auf die *wahrgenommene Nützlichkeit* der Grundfunktionen von *Microsoft Teams* bewerten die Befragten die Funktionen Chat, Voice over IP und Audio‑, Video‑, Webkonferenzen überwiegend *„sehr zufriedenstellend“*. Zu der Portal-Funktion, die zum Zeitpunkt der Befragung noch nicht vollumfänglich bereitgestellt war, gab lediglich eine Person an, einen Nutzen in der Portal-Funktion zu sehen und diese zukünftig zu verwenden. Ebenfalls wird das Mitarbeiterverzeichnis mit integriertem Organigramm mehrheitlich als nützlich angesehen, während eine Person diese Funktion nicht bemerkt hat: *„Die haben sie natürlich auch schon wieder etwas versteckt. (…). Wenn es da einen extra Tab auf der Seite gäbe, wäre mir die Funktion auch aufgefallen.“* Die Befragten bewerten die Möglichkeit für das Erstellen von Tags grundsätzlich als positiv, verwenden diese Funktion im täglichen Gebrauch jedoch eher selten. Das Dokumentenmanagement zum Verwalten und Bearbeiten von Dateien wurde von keiner Person als nützlich erachtet und lediglich sporadisch genutzt. Die mobile App-Version von *Microsoft Teams* haben drei befragte Personen bereits genutzt und empfinden diese als gute Möglichkeit sich über das private Endgerät bezüglich bevorstehender Termine zu informieren oder an Meetings teilzunehmen, wenn sie gerade unterwegs sind. Die Nützlichkeit von Funktionen, die das kollaborative Arbeiten ermöglichen, wird vorwiegend positiv bewertet. Das Teilen des Bildschirms mit Kollegen und gemeinsame Arbeiten werden eher von Softwareentwicklern verwendet. *Scrum*-Master oder Tester arbeiten eher selten in Kollaboration. Nachdrücklich negativ angemerkt wurde die fehlenden Funktionen wie die Zeichenfunktion oder Annotation in *Microsoft Teams*, wie diese Aussage verdeutlicht: *„(…) wenn ich mit anderen Leuten chatte und sie den Bildschirm teilen, fände ich es cool wenn ich da auf dem Bildschirm malen könnte. Das können quasi alle anderen Tools und das benutze ich auch sehr häufig und finde es sehr praktisch. Aber Teams kann das irgendwie nicht.“*. Als weitere fehlende Funktionen wurden die firmenexterne Telefonie oder die Aufnahmefunktion genannt. Auch die Integration von Drittanbieter-Systemen, wie *Jenkins*, wird als nützlich erachtet.

Die vom Benutzer *wahrgenommene Performanz* des Systems beeinflusst auch die Leistungserwartung, denn lange Wartezeiten beeinträchtigen den Nutzen. Die Performanz wird bei den Kernfunktionen Chat 1:1, Chat 1:*n*, Voice over IP und Audio‑, Video‑, Webkonferenz abgefragt. Die Performanz von Einzelchats und Voice over IP wird überwiegend als sehr positiv bewertet. Im Interview kam jedoch auch Probleme bezgl. der Performanz bei größeren Nachrichten zur Sprache: „*Wenn es größere Nachrichten hat, merkt man schon, dass es ein wenig langsamer wird und auch wenn man weit im Chatverlauf hochscrollt, merkt man auch, dass je weiter man im Chatverlauf hochscrollt, es immer schlechter wird von der Performanz.“*.

Die *wahrgenommene Stabilität* der Software ist ebenfalls ein Indikator der Leistungseinschätzung, denn der Abbruch des Systems führt häufig zu Frustrationen der Anwender. Die Stabilität wurde bei den Kernfunktionen Chat 1:1, Chat 1:*n*, Voice over IP und Audio‑, Video‑, Webkonferenz abgefragt. Zu Beginn der Testphase kam es vereinzelt noch zu Systemabbrüchen während der Verwendung. Im Laufe der Testphase wurde das System stabiler und die Stabilität wird von dem Großteil der befragten Personen als positiv wahrgenommen. Lediglich für dieselbe Person mit den Problemen bei der Performanz ist die Stabilität von *Microsoft Teams* nicht zufriedenstellend: *„Ja, bei mir ist Stabilität definitiv der größte Kritikpunkt. Stabilität und das schließt für mich dann auch ein, dass ich Bilder oder Medien, also irgendwelche im Chatverlauf aufgetauchten Screenshots oder sowas, die sehe ich dann halt nicht.“ *Hierzu spekulierte die betroffene Person auf die Netzwerkverbindung als mögliche Fehlerquelle hinter der fehlenden Stabilität.

### Aufwandserwartung

Die Aufwandserwartung fasst alle Aussagen zusammen, die den Grad des Aufwands beschreiben, der mit dem Einsatz und der Benutzung des Systems verbunden ist. Diese Kategorie wird unterteilt in wahrgenommene Einfachheit des Einsatzes und der Interaktion mit dem System. Als *wahrgenommene Einfachheit des Einsatzes des Systems* wurden alle Aussagen kodiert, die eine Einschätzung der befragten Person über die Installation und Konfiguration sowie auch Aussagen über den Aufwand zum Erlernen der Nutzung des neuen Systems wiedergeben. Dazu wurden 19 Textstellen kodiert. Hinsichtlich der Installation von *Microsoft Teams *erklärten die befragten Personen, die das Betriebssystem *Windows10* verwenden, dass seitens der IT-Abteilung ein automatisches Update für *Microsoft Teams* erfolgte und sie somit keinen Installationsaufwand hatten. Für MacOS-Benutzer stellte die selbstdurchgeführte Installation ebenfalls keine Probleme dar. Dagegen wurden die Konfigurationsmöglichkeiten von einigen befragten Personen kritisch gesehen. Der grundlegende Einstieg in *Microsoft Teams* fiel allen befragten Personen leicht, unabhängig davon, ob sie bereits vorher Erfahrungen mit *Microsoft Teams* gemacht hatten oder nicht.

Die *wahrgenommene Einfachheit der Interaktion mit dem System* fasst die Aussagen zur tatsächlichen Benutzung des Systems und des empfundenen Aufwands bei der Interaktion zusammen. Dazu wurden 38 Textstellen kodiert. Die Interaktion mit dem System bei Audio‑, Video-, und Webkonferenzen wird grundsätzlich positiv bewertet. Auch die Voice over IP-Funktion wird als wenig aufwändig bewertet. Die Übersichtlichkeit der Einzel- und Gruppenchats wird als störend empfunden, wie folgende Aussage zeigt. *„Da habe ich hunderte Chats. Mit allen Kombinationen. Und jedes Meeting gibt nochmal einen Chat da rein. Keine gute Übersicht.“*. Ebenfalls wurde bemängelt, dass die System-Einstellungen nicht intuitiv zu finden sind: *„Ich habe lange Zeit geglaubt, es gäbe überhaupt keine Einstellungen in Teams.“ *Diejenigen, die Erfahrungen mit dem System *Slack* gemacht haben, ziehen auch einen Vergleich zwischen den beiden Systemen: *„Vom Umfang und der Bedienerfreundlichkeit finde ich jetzt Slack besser.“*

### Sozialer Einfluss

Die Kategorie des sozialen Einflusses bildet die Beeinflussung durch Normen, Werten und Bezugspersonen im Arbeitsumfeld ab. Dazu konnten nur wenig Textstellen kodiert werden. Lediglich die Aussagen zwei befragter Personen weisen auf die Vorbedingung hin, dass das gesamte Team bevorzugt *Microsoft Teams* für Meetings verwenden soll, und dass damit ein sozialer Einfluss auf den Einzelnen besteht: *„(…) es ist eines der Haupt-Kommunikationstools, die wir hier nutzen*. *Tatsächlich mit allen anderen im Team außer (Name_1) benutze ich Teams.“*. Dennoch wurde auch ein abweichendes Verhalten beobachtet: Eine Person gab an, trotzdem bevorzugt *Slack* für die teaminterne Kommunikation zu nutzen.

### Erleichternde Bedingungen

Erleichternde Bedingungen fassen die Einschätzung der Nutzer, dass sowohl organisatorische als auch technische Gegebenheiten zur Nutzungsunterstützung vorhanden sind. Diese Kategorie wird in die Unterkategorien Infrastruktur und Support unterteilt. In die Unterkategorie Infrastruktur wurden kodierte Einheiten zusammengefasst, die eine Einschätzung zur organisatorischen und technischen Infrastruktur zur Unterstützung von *Microsoft Teams *beinhalten. Keiner der befragten Personen gab grundlegende infrastrukturelle Probleme an. Lediglich die Netzwerkverbindung konnte in manchen Fällen Auswirkung auf die Performanz oder Stabilität haben.

Die Unterkategorie *Support* beinhaltet die Hilfe-Maßnahmen bei Störfällen sowohl seitens der IT-Abteilung bei der Cuvox AG, die für die Integration und Aufrechterhaltung der IT-Infrastruktur zuständig ist, als auch seitens des Systemanbieters. Ein mehrheitliches Feedback der Befragung war, dass kein Support für die Installation und Verwendung von *Microsoft Teams* benötigt wurde. Eine Person gab an, dass eine Auflistung der verschiedenen Funktionen und deren Handhabung seitens des Supports sinnvoll wäre: *„Da wäre es vielleicht mal gut einen Überblick zu haben, was das Tool eigentlich anbietet. Aber jetzt nicht im Sinne von einer Schulung, wie bediene ich das Tool, sondern wirklich einfach ein Überblick über die Möglichkeiten.“*.

### Hedonistische Motivation

Diese Kategorie zeigt an, wie viel Freude bzw. Spaß der Befragten bei der Nutzung des Systems empfinden. Die Ausprägung kann positiv, negativ oder neutral sein. Es wurden einige Aussagen dieser Kategorie zugeordnet. Jedoch ist ein Spaßfaktor bei der Verwendung nicht zu identifizieren. Die Befragten sind glücklich darüber, dass das System stabil läuft und einige nützliche Funktionen bietet. Die Störfaktoren, die bei der Aufwandserwartung erwähnt wurden, wirken sich teilweise auf die hedonistische Motivation der Benutzer von *Microsoft Teams* aus, wie diese Aussage zeigt: *„Ich würde sagen. 70* *% gerne. Es funktioniert und das ist super. Und das ist das wichtigste. Ich habe vorhin schon über die Übersicht meiner Chats geklagt. Das stört mich, das sind ungefähr die 30* *%.“*.

### Gewohnheit

Das Konstrukt der Gewohnheit gibt den Grad an, zu dem Nutzer ein bestimmtes Nutzungsverhalten, welches sie in der Vergangenheit erlernt haben, automatisch ausführen. Hierzu wurden keine Unterkategorien gebildet. Es konnten nur drei Aussagen dieser Kategorie zugeordnet werden. Die Aussagen lassen auf ein mittlerweile gewohntes Verhalten mit *Microsoft Teams* schließen, da alle Befragten angeben, dass sie *Microsoft Teams *häufig nutzen. Einige Personen ziehen den Rückschluss, dass sie das Bedienkonzept von *Slack* durch die jahrelange Nutzung verinnerlicht haben.

### Verhaltensabsicht

Die Verhaltensabsicht gibt die Nutzungseinstellung an. Hierzu wurden zwei Ausprägung zur Kategorisierung gebildet: pro und contra. Die Auswertung der Frage nach dem präferierten Kollaborationssystem für die Zukunft ergibt ein geteiltes Ergebnis. Während drei der Befragten zukünftig eher das System *Slack* nutzen wollen, geben drei andere Personen an, dass sie schon während der Testphase von *Microsoft Teams* überzeugt waren und es zukünftig als Hauptkommunikations-System nutzen würden. Zwei weitere Personen wollen für die interne Kommunikation *Slack* und für externe Kommunikation und Meetings *Microsoft Teams *nutzen. Der große Teil der befragten Personen schildert jedoch den Wunsch nur ein System zu verwenden, wie die nachfolgende Aussage exemplarisch zeigt. *„Also, es wäre schön, wenn es das eine zentrale Tool wäre zur Kommunikation. Ist ein bisschen mühsam mit drei Tools die irgendwie so gleichberechtigt sind“*.

### Nutzungsverhalten

Das Nutzungsverhalten zeigt die tatsächliche Häufigkeit der Nutzung des Systems an. Hierzu wurden drei Ausprägung zur Kategorisierung gebildet: häufige Nutzung, seltene Nutzung und gar keine Nutzung. Aus den Interviews geht hervor, dass *Microsoft Teams* von allen Befragten regelmäßig am Tag verwendet wird, wie dies eine Person exemplarisch betont: *„Jeden Arbeitstag und ja mehrmals pro Arbeitstag. Sicher so 5‑ bis 15-mal pro Arbeitstag.“.* Es wird auch erwähnt, dass das System als Hauptkommunikationssystem verwendet wird: *„Jeden Tag, mittlerweile. Als Haupt-Medium zum kommunizieren mit dem Team und der Firma.“*. Vereinzelt verwenden einige Personen noch *Slack* zur Kommunikation mit anderen Teams oder Fachbereichen: *„(…) teilweise nutzen auch noch andere Kollegen den Team-Channel in Slack.“.*

Schließlich soll auch erwähnt werden, dass die bislang erwähnten Effekte der betrachteten Determinanten der Verhaltensabsicht und Nutzungsverhalten gemäß dem UTAUT‑2 Modell durch verschiedene Moderatorvariable (Geschlecht, Alter, Erfahrung) beeinflusst werden. Die Erfahrungen der Befragten mit anderen Kollaborationssystemen (wie *Slack, Skype for Business* oder *Zoom*) sind bereits erwähnt worden. Dagegen lässt die geringe Anzahl der Befragten (im Alter von bis 25–50 mit einer weiblichen Person) keine zuverlässigen Aussagen über den Einfluss von Alter und Geschlecht zu.

## Diskussion und Fazit

Ziel dieser Arbeit war es, die Akzeptanz von *Microsoft Teams* zur Kommunikation in standortverteilten agilen Entwicklungsteams bei der Cuvox AG zu untersuchen, um zu verstehen, wie Mitarbeiter mit den Funktionen von *Microsoft Teams* umgehen, welche Funktionen akzeptiert bzw. nützlich erachtet werden und welche nicht. Außerdem sollte evaluiert werden welche zukünftige Akzeptanzeinstellung sich gegenüber *Microsoft Teams* etabliert hat. Die Ergebnisse sollen nun auf die Fragestellung der Studie hin zusammenfassend diskutiert und Implikationen für die Forschung und Praxis abgeleitet werden.

### Interpretation der Ergebnisse und Beantwortung der Forschungsfragen

Nach UTAUT-2-Modell hängt das *Nutzungsverhalten* von Verhaltensabsicht und weiteren Faktoren ab. Die Daten zum *Nutzungsverhalten *zeigen, dass *Microsoft Teams *für die teaminterne Kommunikation regelmäßig eingesetzt wurde. Vor allem wurden Einzel- und Gruppenchats sowie auch die Funktion der Videokonferenz für Meetings verwendet. Zusätzlich nutzten einige Personen das System für teamexterne Kontakte (z. B. mit Kunden). Für dieses Nutzungsverhalten spielten zwei wichtige Determinanten wie die Leistungserwartung und Aufwandserwartung eine wesentliche Rolle. Darüber hinaus kann die Wirkung des sozialen Einflusses auf die aktuelle Nutzungsabsicht und damit das aktuelle Nutzungsverhalten in der Testphase- trotz der wenig kodierten Texte – allgemein angenommen werden, da die Nutzung von *Microsoft Teams *die Vorbedingung der Testphase und der Regelung war. Es stellt sich hier die Folgefrage, welche Einstellungen sich dabei zur zukünftigen Nutzung des Systems bei den Befragten gebildet haben.

Die Aussagen zur zukünftigen *Verhaltens- bzw. Nutzungsabsicht* stellen unterschiedliche Absichten dar. Während einige der Befragten sich klar für die zukünftige Nutzung von *Microsoft Teams* aussprechen, bevorzugen einige das System *Slack*. Die Gründe für diese Unterschiede lassen sich durch die nähere Betrachtung der Determinanten der Verhaltensabsicht erklären.

Auf der positiven Seite zeigen die Ergebnisse der *Leistungserwartung*, dass viele Kommunikationsfunktionen von *Microsoft Teams*, wie Chat oder Telefonie, sowie Audio‑, Video-, und Webkonferenzen, als besonders nützlich bewertet wurden, auch wenn es hinsichtlich der Performanz und Stabilität einige wenige Störfälle gegeben hat. Das kollaborative Arbeiten in verteilten Teams mit *Microsoft Teams* wird ebenfalls positiv bewertet. Auf der negativen Seite fehlen hier jedoch einige Funktionen, die nützlich erachtet werden, wie z. B. die Annotationsmöglichkeit (Zeichenfunktion) sowie auch die Anbindung zu Drittanbieter-Software wie *Jenkins*. Bei der Wahrnehmung dieser Defizite spielten die Erfahrungen der Befragten mit anderen Kollaborationssystemen (wie z. B. *Slack*), die diese Funktionen besitzen, eine besondere Rolle.

In Bezug auf die positiven Wirkungen der *Aufwandserwartung* auf die Nutzungsabsicht lassen sich die Einfachheit des Einsatzes des Systems, vor allem die Installation von *Microsoft Teams*, sowie auch der grundsätzlich als intuitive wahrgenommene Einstieg als besondere Eigenschaften hervorheben. Auf der anderen Seite sind zu erwähnen, dass einige Befragten die Konfigurationsmöglichkeiten bemängelten und mehr Wert auf die Einfachheit der Konfiguration als die Einfachheit der Installation als einmaliger Vorgang gelegt haben. Außerdem wurde die Benutzerfreundlichkeit der Interaktion mit dem System – im Vergleich zu *Slack* – von vielen als deutlich negativer angesehen. Vor allem die aufgrund des Funktionsumfangs überladene Menüstruktur (mit Funktionen, die nicht verwendet werden) führt zu Navigationsaufwand, der als zunehmend störend empfunden wird. Der Wunsch nach einer einfachen Interaktion mit dem System ist erkennbar. Insgesamt lassen die Daten in Bezug auf die Wirkung der Aufwandserwartung auf die Verhaltensabsicht – im Einklang mit dem UTAUT-2-Modell – Folgendes erkennen: Die Befragten mit der klaren Absicht *Microsoft Teams* zukünftig weiterzuverwenden, bewerteten zuvor die wahrgenommene Einfachheit des Einsatzes und der Interaktion mit dem System meist positiver als diejenigen, die lieber zukünftig *Slack* verwenden wollen. Dabei spielten die positiven Erfahrungen mancher Befragten mit der Bedienung des Vergleichssystems *Slack* eine besondere Rolle, nämlich, dass diese Erfahrungen zu einer Verstärkung des Aufwandsgefühls von *Microsoft Teams* geführt haben. Die Argumente für *Slack* liegen momentan noch auf der Gewohnheit und eingespielten Prozessen. Schließlich sollte an der Stelle erwähnt werden, dass *Slack* in *Cuvox AG* langfristig nur noch eingeschränkt genutzt werden kann, da nur die kostenlose (Free-Version) mit beschränken Optionen (Anrufe nicht mehr möglich etc.) genutzt werden soll. Dies kann eventuell zur Folge haben, dass die Nutzungsabsicht abnimmt und Microsoft Teams sich als zentrales Kommunikationstool etabliert.

In Bezug auf den Einfluss der *erleichternden Bedingungen* (Infrastruktur und Support) auf das Nutzungs- und Akzeptanzverhalten zeigen die Ergebnisse eine positive Wirkung. Für die Nutzung von *Microsoft Teams* als ein Cloud-basiertes System ist eine ständige und stabile Netzwerkverbindung (auch im privaten Umfeld aufgrund vermehrter Home-Office-Tätigkeiten bedingt durch Covid-19-Pandemie) essenziell. Auf die privaten infrastrukturellen Rahmenbedingungen hat die *Cuvox AG *wenig Einfluss. Es liegt im eigenen Interesse der Mitarbeiter die Rahmenbedingungen für ihr Home-Office einzurichten. Die firmeneigenen Netzwerke wurden an allen Standorten als stabil wahrgenommen. Es wurde auch ersichtlich, dass die Erfahrung der Interviewteilnehmer, die Inanspruchnahme des Supports für den Umgang mit *Microsoft Teams* obsolet macht. Bevorzugt gaben Softwareentwickler an, dass der Anspruch besteht, dass eine Software wie *Microsoft Teams* keine unterstützenden Tätigkeiten seitens des Supports bedarf. In Bezug auf den Einfluss der erleichternden Bedingungen auf die Akzeptanz von *Microsoft Teams* lassen sich daher gute infrastrukturelle Bedingungen (ohne den Bedarf eines Supports) als positiv wirkende Faktoren aus dieser Studie ableiten.

Im Hinblick auf die Wirkung des *Sozialen Einflusses *wurde ein Einfluss auf das aktuelle Nutzungsverhalten in der Testphase angenommen, da die Nutzung von *Microsoft Teams* die Vorbedingung der Testphase und der Regelung war. Die jeweiligen Teammeetings wie *Daily Scrum, Retrospektive* oder *Planning* wurden mit *Microsoft Teams *durchgeführt. Jedoch lassen die sehr wenig kodierten Textstellen keine Aussagen über die Wirkung auf die zukünftige Verhaltensabsicht zu. Dennoch kann allgemein – und basierend auf den bisherigen Erkenntnissen der Akzeptanzforschung – von einem sozialen Einfluss auf zukünftige Nutzungseinstellungen und Nutzungsverhalten unter den sozialen Bedingungen der zukünftigen Nutzung ausgegangen werden.

Der Einfluss von *Gewohnheit* auf die Einstellung und das Nutzungsverhalten konnte trotz wenig kodierter Textstellen beobachtet werden. *Microsoft Teams *wird mittlerweile sehr häufig genutzt, was auf eine Gewöhnung an das System schließen lässt. Jedoch ist – wie bereits erwähnt – durch die jahrelange Nutzung von *Slack *noch immer eine Gewöhnung an dessen Bedienkonzept zu erkennen. Wenn auch eine Routine im Umgang mit *Microsoft Teams *an manchen Stellen noch nicht erkennbar ist, kann davon ausgegangen werden, dass eine langfristige Nutzung von *Microsoft Teams *hier zu einer Besserung führt, mit möglichen positiven Folgen für die Einstellungen und Nutzungsverhalten von zukünftigen Nutzern.

Schließlich kann auch der Einfluss der *hedonistischen Motivation* beim Umgang mit *Microsoft Teams* nicht zweifelsfrei festgestellt werden. Viele Befragten betrachteten das System ein Mittel zum Zweck, und der Spaß stand für sie nicht im Vordergrund bei der Anwendung. Lediglich lässt sich bei einigen der Befragten indirekt negative Einfluss auf die hedonische Motivation durch die negativen Erfahrungen bezgl. der Benutzerfreundlichkeit (wie bereits erwähnt) annehmen.

### Limitationen und Implikationen für Forschung und Praxis

Um den Rahmen und Bedingungen der Entstehung und Interpretation der Ergebnisse transparent zu machen, sollten an der Stelle auch die Limitationen der Arbeit erwähnt werden. Zum einen betrifft diese die Stichprobengröße und damit einhergehende Einschränkung, die Einflüsse der Moderatorenvariablen wie Geschlecht und Alter zu betrachten und auch die Erkenntnisse der Studie zu generalisieren. Zum anderen lassen die teilweise nur wenig kodierten Aussagen zu manchen Kategorien (wie dem sozialen Einfluss, der hedonistischen Motivation und Gewohnheit) noch Fragen offen.

Diese Limitationen geben auch Anlass und Motivation für weitere Forschung. Zum einen können ergänzende qualitative Fallstudien in weiteren Anwendungs- bzw. Unternehmenskontexten die Vergleichbarkeit von Erkenntnissen ermöglichen. Zum anderen kann eine quantitative Studie mit größer Stichprobe nicht nur zuverlässige Generalisierungen von Ergebnissen sichern, sondern auch die Betrachtung verschiedener Aspekte (wie z. B. Einflüsse von Moderatorenvariablen) einschließen, die hier aufgrund der Stichprobengröße nur am Rande berücksichtigt wurden. Im Allgemeinen erweist sich die Kombination qualitativer und quantitativer Forschungsmethoden im Sinne eines Mixed-Methods-Ansatzes als eine geeignete Strategie, um einen ganzheitlichen und umfassenden Einblick in technische, soziale und organisationale Rahmenbedingungen und individuelle Bedürfnisse zu gewinnen (Schreier und Odağ 2020). Darüber hinaus können motivationale Aspekte der Nutzung von Microsoft erforscht werden. Hierzu bieten sowohl empirische als auch verschiedene gestaltungsorientierte Ansätze Orientierung, um beispielweise Motivation und Werte im Zusammenhang zu betrachten (vgl. Yetim 2011; Yetim et al. 2011) und verschiedene persuasive Designstrategien (vgl. Oinas-Kukkonen und Harjumaa 2009; Yetim 2013) im Kontext von *Microsoft Teams* zu berücksichtigen.

Die Ergebnisse haben auch Implikationen für die Praxis. Erstens stellen sich auch in dieser Studie die Leistungserwartung und Aufwandserwartung – im Einklang mit dem UTAUT-Modell- als wichtige Faktoren heraus, die bei der Implementation von *Microsoft Teams* berücksichtigt werden sollten. Um den identifizierten negativen Wahrnehmungen hinsichtlich der Benutzerfreundlichkeit der Interaktion entgegenzuwirken und auch dem Wunsch nach mehr Fokus auf die Kommunikationsfunktionen entgegenzukommen, bieten sich verschiedene Lösungsmöglichkeiten an. Zum einen kann der Funktionsumfang von *Microsoft Teams* (jedenfalls für den Entwicklungsbereich) reduziert bzw. auf die jeweiligen Nutzergruppen angepasst werden. Zum anderen kann durch die Erstellung von Blog-Post-Einträge über die Möglichkeiten von *Microsoft Teams* berichtet werden, um den Funktionsumfang verständlich zu machen. Darüber hinaus sollten weitere Wünsche des Entwicklungsteams nach (a) zusätzlichen Funktionen für kooperatives Arbeiten (z. B. Zeichenfunktion), (b) mehr Konfigurationsmöglichkeiten in *Microsoft Teams* und (c) der Selbstgestaltung der Kanäle erwähnt werden. Im Hinblick auf die beobachteten Stabilitätsprobleme, die sich häufig in der Einführungsphase einer Software beobachten lassen, ist darauf hinzuweisen, dass diese zeitnah von der IT-Abteilung erörtert und behoben werden können, im Sinne der Verbesserung der erleichternden Bedingungen gemäß dem UTAUT-Modell, um die Frustration der betroffenen Personen zu senken. Schließlich lassen sich auch Erkenntnisse aus anderen Studien als handlungsrelevante Einsichten erwähnen, nämlich dass die Einführung neuer Softwaretools und Arbeitsweisen vom einem systematischen Veränderungsprozess begleitet werden sollten. Unter anderem wird dabei für Führungskräfte die Vermittlung von Motivation trotz Distanz durch klare Kommunikation, neue Führungsmethoden und die konstruktive Nutzung von Software als entscheidend angesehen (Lindner 2020), um sowohl rationale als auch emotionale Einsicht zu ermöglichen und Rückfälle in alte Verhaltensweisen zu vermieden.

### Fazit

Zusammenfassend lässt sich festhalten, dass die Ergebnisse dieser Arbeit unterstützen einige Annahmen der Akzeptanzforschung. Die Einstellung zur Nutzung eines Systems und das tatsächliche Nutzungsverhalten werden unter anderem erheblich von der Leistungserwartung (wie z. B. der Performanz, Stabilität und Nützlichkeit), der Aufwandserwartung (wie z. B. der Benutzerfreundlichkeit der Interaktion mit dem System) beeinflusst. Die Untersuchung macht auch deutlich, dass ein zentrales System zur Kommunikation und Kollaboration mittelfristig als förderlich betrachtet wird. *Microsoft Teams* bietet mit der Integration in die Microsoft-Systemlandschaft langfristig mehr Möglichkeiten. Diese Studie legt gewisse Einflussfaktoren, Stärken und Schwächen aus der Sicht eines Entwicklungsteams offen, um Möglichkeiten zur Steuerung bzw. Beeinflussung der Akzeptanz anzubieten. Es sollte jedoch zum Abschluss betont werden, dass die empirischen Ergebnisse einer Studie Momentaufnahmen sind, und dass die Schwächen eines Systems mit fortschreitender Zeit behoben werden. Wie sich das hier untersuchte System *Microsoft Teams* weiterentwickelt, bleibt abzuwarten.
